# Hazard perception in manual and hands-off Level 2 driving under daytime and after dark lighting conditions: A driving simulator study

**DOI:** 10.1007/s10111-026-00870-9

**Published:** 2026-05-19

**Authors:** Yee Thung Lee, Rafael C. Gonçalves, İbrahim Öztürk, Albert Solernou, Karl Proctor, Natasha Merat

**Affiliations:** 1 Institute for Transport Studies, University of Leeds, Woodhouse Lane, Leeds, LS2 9JT UK; 2 Jaguar Land Rover, Coventry, UK

**Keywords:** Hazard perception, Daytime driving, After dark driving, Hands-off L2 automation, Manual driving

## Abstract

The aim of this study was to examine drivers’ hazard perception in an urban scene, under two different lighting conditions (daytime and after dark) and for two different levels of driving automation (manual driving and hands-off SAE level 2 automation). Forty-eight participants took part in four experimental drives in a driving simulator, encountering six different hazardous/potentially hazardous events in each drive. Results showed that drivers detected hazards significantly earlier and were more likely to react to the hazards in daytime, compared to after dark environments, particularly when pedestrians were approaching the road from the left. However, there was no significant difference in their response time towards the hazards, between the daytime and after dark environments. In terms of driver response in the two automation levels, the majority of drivers were proactive and reacted before the potentially hazardous events turned into actual hazards during manual driving, but responses were more reactive during automated driving. These findings highlight the need to account for context of the driving environment such as lighting conditions and levels of driving automation when designing systems or protocols that aim to support hazard perception and timely driver response.

## Introduction

According to a report by the National Highway Traffic Safety Administration (NHTSA 2023), 17.83% more fatal crashes happen on the road during after dark hours, when compared to daytime hours. Raynham et al. (2020), analysed the traffic collision statistics in the United Kingdom and found a 19.3% increase in crashes in after dark compared to daytime conditions. Crashes at night are typically based on a combination of human and environmental factors. Examples of human error associated with driving after dark include risky driving behaviour, especially for young drivers (Clarke et al. 2005), driving under the influence of alcohol (Williams 2003) or opting to drive despite experiencing drowsiness (Dawson et al. 2021; Lowden et al. 2009). Environmental factors include reduced visibility and limited sight distance at night (Anarkooli and Hosseinlou 2016), leading to delayed reactions to potential obstacles.

However, despite the above findings, results are mixed when considering the effect of driving after dark on vehicular control. For example, regarding speed control, an on-road study by De Valck et al. (2006) showed that drivers drove faster after dark compared to daytime conditions, which the authors suggest is an intentional effort to stay alert. In contrast, Sandberg et al. (2011) found that drivers adopt compensatory behaviours after dark by driving slower, which the authors suggest is based on an intentional effort to maintain safety. Finally, Li et al. (2022) found that drivers had lower odds of speeding and recognised more road signs in after dark conditions, suggesting heightened attention and vigilance, when compared to daytime conditions. It is noteworthy that the participants from Li et al. (2022) had a lower mean age (28 years old) compared to those in De Valck et al. (2006) and Sandberg et al. (2011), whose participants’ mean age were 40 and 41 years old, respectively. Regarding lateral control, a driving simulator study conducted by Öztürk et al. (2023), showed that drivers were worse at controlling the lateral movement of the vehicle after dark, compared to daytime conditions, which they suggest is due to the low visibility of the driving environment. On the other hand, real-world studies report that drivers tend to drive closer to the midline after dark, to prevent colliding with any adjacent obstacles (Sandberg et al. 2011).

Drivers not only adapt their vehicular control when driving after dark, but also alter their visual sampling behaviour, which can ultimately influence their hazard perception (HP). HP is defined as drivers’ ability to detect, evaluate and respond to any on-road events that are likely to lead to a crash (Crundall et al. 2012). A review of real-world studies showed that drivers exhibit a narrower gaze dispersion when driving after dark, when compared to daylight conditions (Grüner and Ansorge 2017). This can result in a reduction of their peripheral HP, such as noticing pedestrians attempting to cross the road. This change in visual sampling behaviour when driving after dark due to low visibility can lead to a potential delay in perceiving hazards, impairing HP.

To date, only a limited number of studies have investigated drivers’ HP after dark (Asadamraji et al. 2019; Evans et al. 2022; Garay et al. 2004; Garay-Vega et al. 2007). For example, a computer-based experiment (Evans et al. 2022), showed that drivers identified significantly fewer hazards after dark, compared to daytime, conditions. Garay et al. (2004) and Garay-Vega et al. (2007) also suggest that drivers fixated on the potentially hazardous regions more often in the daytime, compared to after dark, conditions. Based on data collected from a driving simulator study, Asadamraji et al. (2019) showed that HP reduced significantly in after dark condition for experienced drivers, and even more for novice drivers (35% and 67% lower than daytime condition, respectively). Finally, Plainis and Murray (2002) showed that low luminance at night can reduce drivers’ reaction time to hazards.

Most of the work on HP at night has focused on manual driving, with little known about how HP is affected by automated driving. In manual driving, drivers are required to remain in the control loop and are responsible for both the lateral and longitudinal control of the vehicle (Merat et al. 2019). Such continuous involvement of the motor control loop allows perceptual-motor coupling to remain intact, guiding the trajectory of the vehicle (Mole et al. 2019). In addition, to execute operational steering and pedal actions, drivers need to regularly shift their gaze between the far-road and near-road regions (Land and Horwood 1995), to gather the necessary visual information for lateral and longitudinal control of the vehicle. Mackenzie and Harris (2015) suggested that removing drivers from operational controls (lateral and longitudinal) could reallocate freed-up attentional resources by enabling them to scan the driving environment more extensively (i.e., higher horizontal gaze dispersion).

With the introduction of automated vehicles, drivers can relinquish some or all of their responsibility to the automated system. For instance, in Partial Driving Automation (level 2; SAE 2021), the vehicle is equipped with an Advanced Driving Assistance System (ADAS) that keeps the vehicle in its driving lane (Lane Keeping Assistance; LKA), adhering to the posted speed limit, while maintaining a designated headway from a lead vehicle (Adaptive Cruise Control; ACC). One of the key challenges that emerges during automation is that when drivers are no longer responsible for operational control, particularly lateral control, the perceptual-motor coupling involved in steering breaks down, because gaze direction no longer informs the limbs to control the trajectory of the vehicle (Mole et al. 2019). Navarro et al. (2016) compared drivers’ steering control and eye movements during an obstacle avoidance task, for an automatic steering (AS) condition and manual driving. Results found more look-ahead fixations in the AS conditions, when compared to the manual drives. Drivers also demonstrated more unstable driving after taking over manual control, indicated by a higher maximum lateral deviation and lower time-to-collision (TTC). Therefore, disruption of visuomotor coordination, brought about by the AS condition, potentially influenced drivers’ performance in obstacle avoidance. It can be argued that this disruption of perceptual-motor coupling during automation could also impair drivers’ response to hazards, and this could potentially be even more exacerbated if drivers have their hands-off the steering wheel in hands-off L2 automation, which was the focus of the study.

Although there were no official guidelines on SAE documents on whether L2 automation was hands-on or hands-off, the current L2 vehicles that are more prevalent on the market are hands-on. However, car manufacturers were exploring the potential of having hands-off L2 driving systems (ETSC 2023). Although hands-off L2 automation is currently restricted to certain traffic situations (i.e., motorways), it can be considered a transitional stage between SAE L2 and SAE L3. Therefore, it is essential to investigate whether complete breakage of physical control, when the driver’s limbs are not in contact with the vehicle controls (both the steering wheel and the pedals) during automation, influences their HP (both detection and reaction stages). For example, a recent study showed that drivers had quicker reaction times to silent automation failures (i.e., automation failures that are not detected by the system and do not trigger an alert or takeover request) during hands-on L2 than during hands-off L2 (Hussain et al. 2025).

The introduction of automation not only influences drivers’ visual attention allocation, but also their hazard avoidance tactics. Studies have shown that drivers’ response time to obstacles is higher after resumption of control from automation, when compared to manual driving (Blommer et al. 2017; Eriksson and Stanton 2017; Gold et al. 2013). Louw et al. (2015) also showed that drivers initiate more evasive manoeuvres, such as a higher maximum lateral acceleration after resuming control from automated driving, compared to manual driving.

To date, much of the work on driving performance and visual attention in automated driving has focused on the periods around a takeover request, with less work dedicated to understanding drivers’ proactive HP, in the absence of takeover alerts. Overall, driving simulator studies have not found any major differences in HP between manual and L2 automated driving (see Greenlee et al. 2024; Samuel et al. 2020; Zangi et al. 2022). For example, Greenlee et al. (2024) measured drivers’ HP during a 40-min period, asking them to press a button on the steering wheel when they detected hazards. Although no significant difference in HP was seen between L2 and manual driving, the magnitude of decrement in HP was significantly higher as the drive progressed in L2, when compared to manual driving. Specifically, as the drive progressed in L2, drivers had an increased likelihood of false alarms and impaired ability in discriminating between hazardous and non-hazardous events. One shortcoming of this work, however, is the use of a button for detecting hazards, as opposed to actual response to hazards, with real driving-based responses.

To the best of our knowledge, there is also a lack of understanding of how HP is affected by different lighting conditions of the driving environment, for manual and automated driving. For example, the additional demand associated with driving after dark may potentially interact with the different demands introduced by the automated driving system, which remains to be explored. Wood (2020) argued that reduced illumination when driving after dark was one of the major contributory factors associated with heightened crash risk due to humans’ visual performance deteriorating in terms of visual acuity and contrast sensitivity. It was postulated that this reduced visual performance could impact HP when driving after dark. It has been argued that engagement of automated driving systems relieves drivers from the moment-to-moment operational control of the vehicle (Mole et al. 2019), releasing mental resources which can then be used for more efficient scanning of the driving environment (Mackenzie and Harris 2015). Therefore, it can be argued that when driving with hands-off L2 after dark, the availability of these additional resources will allow faster detection of hazards, compared to manual driving. Consequently, the effect of lighting conditions on HP may not be uniform across levels of driving automation, resulting in potential interaction effects. For this reason, this study focused on investigating the effect of lighting of the external driving environment (daytime and after dark) and level of driving automation (manual and hands-off L2 driving) on HP with the following research questions:

1. What is the effect of lighting of the external driving environment and the level of driving automation on drivers’ attention to both actual (materialised) and potential (non-materialised) hazards?
2. What is the effect of lighting of the external driving environment and the level of driving automation on drivers’ reaction to both actual (materialised) and potential (non-materialised) hazards?

Based on previous studies, it is hypothesised that drivers would have worse HP (both detection and reaction) after dark compared to daytime conditions. Given the reduced attentional demands required for operational controls during hands-off L2 automated driving, it is hypothesised that drivers would be quicker to detect hazards during hands-off L2 than during manual driving. However, for reaction to the hazards, as drivers would be constantly in control of the movement of the car during manual driving, drivers would have better performance in reacting to the hazardous events. For the interaction effects, it is hypothesised that the effect of automation would be modulated by the effect of lighting conditions of the driving environment for their HP.

## Methods

### Participants

Ethical approval was granted by the University of Leeds Ethics Committee (BESS+FREC 2023-0792-877). A G-power analysis (Faul et al. 2007) was conducted to determine the sample size for a 2 × 2 within-participants experimental design. This suggested that at least 36 participants were required for achieving a well-powered output (1–*β* = 0.95), with a medium effect size (*f* = 0.25), and a significant alpha criterion (*α* = 0.05), for one group of participants with four measurements (correlation among repeated measures = 0.5, and non-sphericity assumed).

To fulfil the G-power requirements and achieve a fully counterbalanced order of experimental conditions across participants, 48 participants (28 male, 20 female) were recruited. They were aged between 25 and 63 years old (*M* = 40.31, *SD* = 11.04), and had normal or corrected-to-normal vision. They were regular drivers who drove at least once a week and held a valid UK driving license for at least three years. Their annual mileage ranged from 3000 to 18,000 miles (*M* = 8125, *SD* = 3440).

### Apparatus

**Fig. 1:**
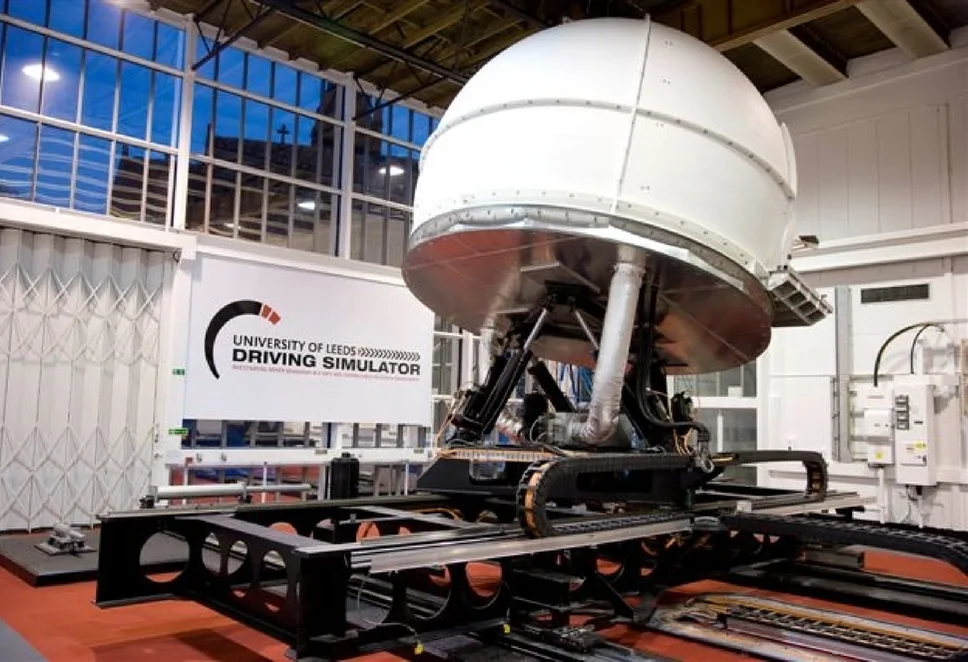
University of Leeds driving simulator (UoLDS)

The experiment was conducted in the University of Leeds Driving Simulator (UoLDS), a high-fidelity motion-based driving simulator that provides a safe and controlled environment for experimental studies (Fig. 1). Placed within a 4-m spherical dome, an S-type Jaguar is equipped with operational pedals and a steering wheel. The dome provides a 300-degree projection to display the driving environment. The UoLDS has an electrical motion system equipped with 8 degrees of freedom, allowing the movement of the dome in six different orthogonal directions.

Tobii Pro Glasses 3 (Tobii AB 2024) were used to capture participants’ gaze data at 50 Hz. The glasses were also equipped with a scene camera that allowed a resolution of 1920 × 1080 pixels video recordings, covering a 106 degrees view from the drivers’ perspective. These recordings were superimposed with the gaze data, allowing post-processed annotation of dynamic Areas of Interest (AoIs), using the Tobii Pro Lab software.

### Design

The experiment was conducted using a 2 × 2 within-participants design, with lighting of the external driving environment and level of driving automation (Hands-off Level 2 Automation and Manual Driving) as the independent variables.

**Fig. 2:**
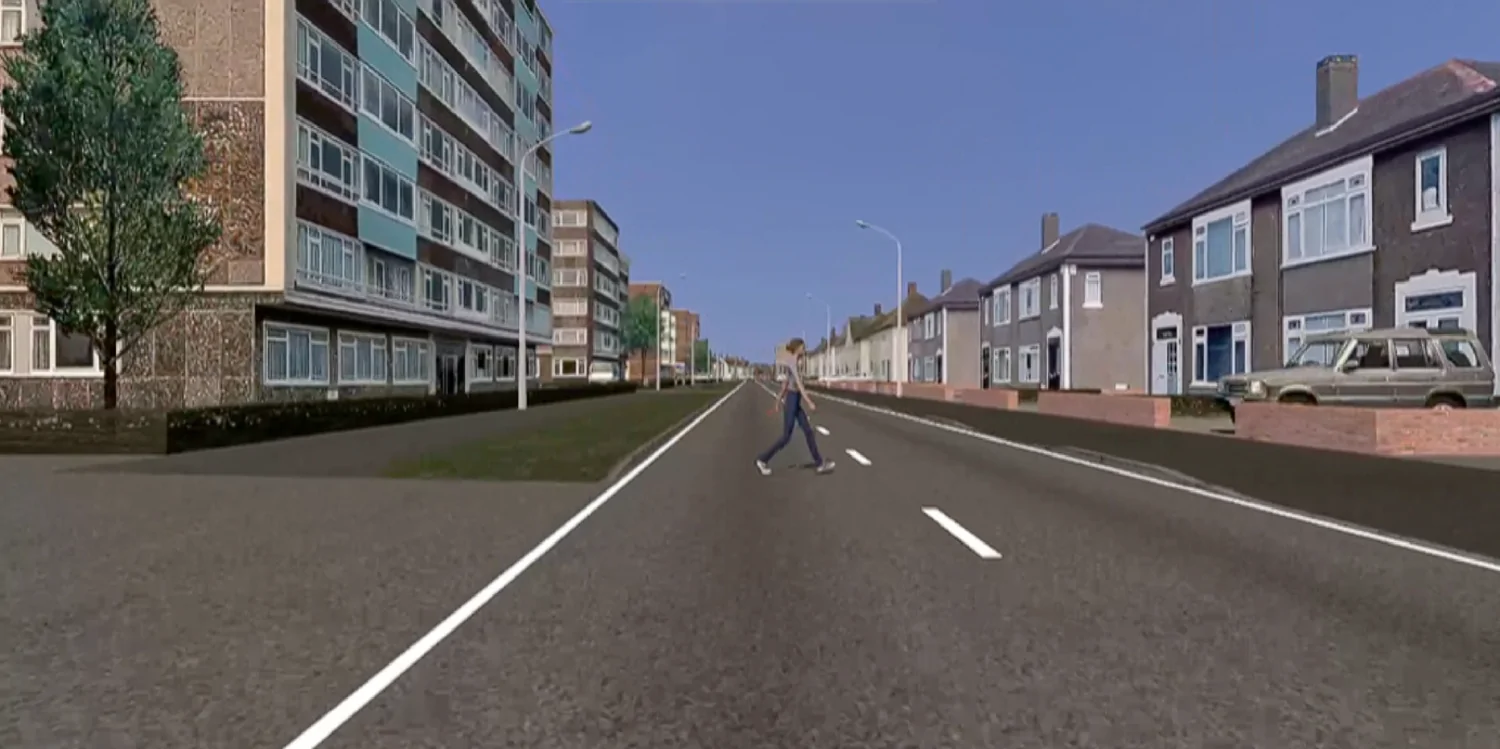
An example of “left pedestrian crossing” event in the daytime driving environment

**Fig. 3:**
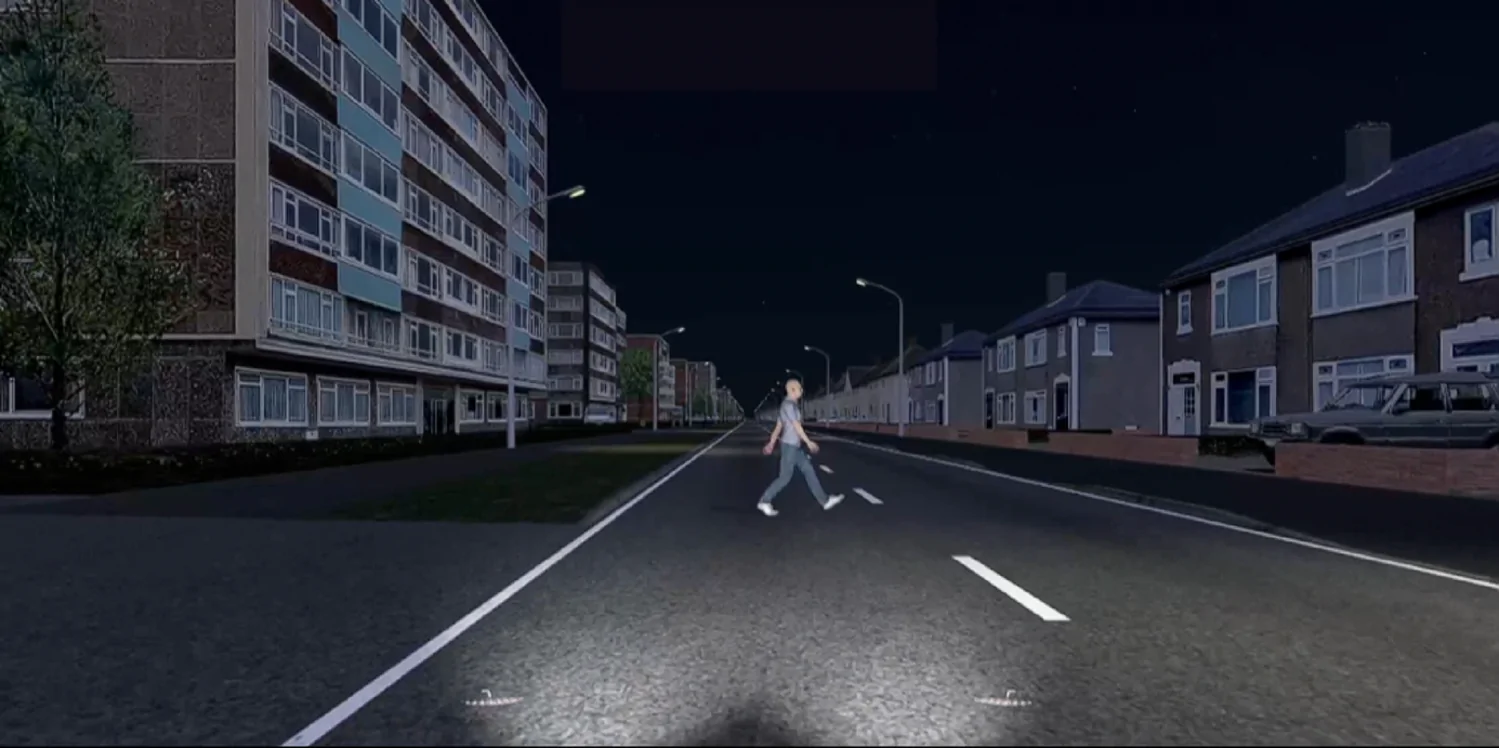
An example of “left pedestrian crossing” event in after dark driving environment

The lighting of the external driving environment was manipulated with the ‘Global Illumination (GI)’ system within Unity. In the Daytime conditions, the ambient illumination was set to be 12:00PM afternoon, whereas in the After Dark conditions, the illumination was set to be 12:00AM midnight with streetlights and the vehicle’s headlights on (see Fig. 2 for Daytime and Fig. 3 for After Dark conditions, respectively). Participants completed four experimental drives in a fully counterbalanced order (one of each combination of conditions), with a short break after every two drives. All testing sessions were conducted during the daytime (between 9AM to 5PM), preventing the potential confounds related to drowsiness or fatigue, ensuring that the present study isolated the effects of lighting conditions of the driving environment and its interaction with level of driving automation on HP.

For the manual drives, participants were instructed to drive a section of urban road as they would in the real world. They were responsible for controlling the vehicle’s lateral and longitudinal movement and were instructed to adhere to the posted speed limits (30 mph).

**Fig. 4:**
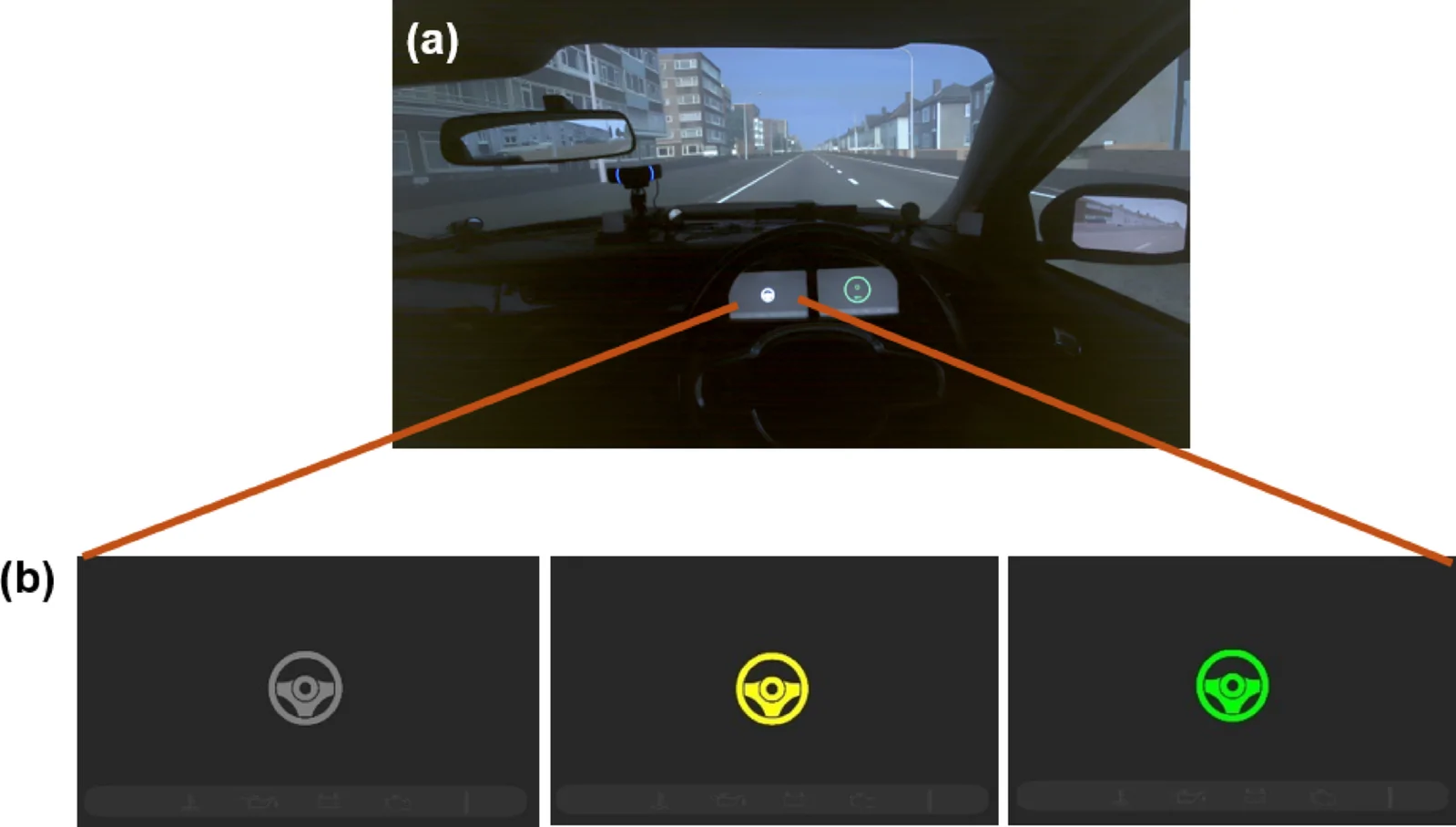
**a** The dash area of the vehicle, showing the human machine interface (left) and current speed (right). **b** The steering wheel icons used to display the automation status: Automation not available (Grey, left), Automation Available (Yellow, middle), and automation engaged (Green, right)

For the level 2 automated drives, the vehicle was equipped with an Advanced Driving Assistance System (ADAS) for speed control and lane keeping. Participants drove the same type of urban environment and were required to keep the automation system engaged as much as possible, whenever it was available. At the beginning of the automated driving conditions, participants first encountered a 1-min period of manual driving, before the system was available. An auditory prompt: “automation available”, was presented by the car’s speakers, accompanied by a change in the colour of the steering wheel icon from grey to yellow. This was located in the dashboard area and represented the automation status (see Fig. 4). When available, participants were required to engage the automation as soon as possible by pressing a button on the steering wheel. The system would automatically engage automation after 10 s, if this button was not pressed. After the system was engaged, the colour of the steering wheel icon changed from yellow to green. Once the system was engaged, the vehicle maintained the posted speed limit of 30 mph and drove in the centre of the lane. Participants were told that the system was not able to detect and respond to any on-road hazards. Therefore, they had to monitor the driving environment at all times and take over manual control in order to avoid a collision. This disengagement was achieved by turning the steering wheel by 2 degrees, pressing the brake pedal, or pressing the same button on the steering wheel. If they disengaged the automation, participants were required to re-engage it as soon as possible, or the system would automatically re-engage after 10 s.

### Hazard perception events and roadway design

Participants travelled along a two-lane urban roadway, populated with different ambient road elements, also involving a range of road users, depicting a typical urban environment. These included parked trucks and cars, walking pedestrians, groups of talking pedestrians, bus stops, zebra crossings and static cyclists. These were equally spaced every 40 m and randomly positioned on the left and right, along the road. The drive also included oncoming cars, travelling at the rate of 1.6 cars per mile.

In line with previous studies on HP (Borowsky et al. 2010), participants encountered six different events for each of the four experimental drives, presented in a randomised order, to reduce learning effects. Three of these events were “materialised” and three were “non-materialised” events. Based on previous work (Ventsislavova et al. 2016), materialised events were defined as actual hazardous events that required initiation of manoeuvres by the driver to avoid a crash, whereas non-materialised events were defined as potentially hazardous events that did not result in a crash, even if the driver failed to reduce the vehicle’s speed or lateral position. Care was taken to ensure the events occurred equally in the forward roadway, on the left and right side of the road, to avoid biased spatial-based attention selection scanning strategies (Carrasco 2011). The hazardous elements also included both pedestrians and cars to avoid biased feature-based attention selection scanning strategies (Most and Astur 2007). The materialised events were left pedestrian, right pedestrian and oncoming car events, whereas the non-materialised had the same hazardous elements but did not develop into actual hazards. The pedestrian approaching from the right walked at a marginally faster rate than those approaching from the left to account for the longer distance along their path. Please see Table 1 for a more detailed description. As each event was different in terms of its criticality and the behaviours involved, six events were analysed separately (see Sect. 2.7 for further information).

**Table 1:** Detailed description of each event in the experimental drives

Materialisation status	Event type	Description	Schematic diagram
Materialised	Left pedestrian	A pedestrian walked from a side alley towards the road (walking speed = 0.75 m/s) and crossed the road from the left of the ego vehicle. This led to a TTC of 2 s	
Materialised	Right pedestrian	A pedestrian walked from a side alley towards the road (walking speed = 1.5 m/s) and crossed the road from the right of the ego vehicle. This led to a TTC of 4 s	
Materialised	Oncoming car	An oncoming car with its right indicator on travelled towards the ego vehicle at a speed of 11 m/s), decelerated at a rate of − 2 m/s^2^, and turned into a side road (turning speed = 3 m/s). This led to a TTC of 2.5 s	
Non-materialised	Left pedestrian	A pedestrian walked from a side alley towards the road (walking speed = 0.75 m/s) and stopped 0.5 m from the edge of the road	
Non-materialised	Right pedestrian	Pedestrian walked from a side alley towards the road (walking speed = 1.5 m/s) and stopped 0.5 m from the edge of the road	
Non-materialised	Oncoming car	An oncoming car with its right indicator on travelled towards the ego vehicle at a speed of 11 m/s), decelerated at a rate of − 2 m/s^2^, and stopped and waited for the ego vehicle to pass	

### Procedure

Upon arrival, participants were briefed with the essential details about the experiment and were allowed to ask any questions, before providing consent to take part in the experiment. After providing consent, participants were required to complete a pre-drive self-report questionnaire to rate their trust and perceived safety of automated vehicles (results are not included in this manuscript).

Participants were then accompanied to the simulator by the experimenter and completed two practice drives (driving in manual mode in an after dark environment and driving in L2 automated mode in a daytime environment). Here, they familiarised themselves with the operational controls of the driving simulator, engaging and disengaging the automation. No hazardous event was included in the practice drives.

During each experimental drive, participants encountered an event (materialised or non-materialised) about every minute, and each complete experimental drive took approximately 9 min. After the last experimental drive, participants completed a post-drive questionnaire, which included questions about their perceived driving skills and any difficulties associated with driving after dark. They were also asked to rate their trust and perceived safety of automated vehicles for a second time (this data is not reported here). Participants were compensated £30 for their time, which was approximately 90 min.

### Data preprocessing

Data preprocessing was conducted using Python 3.9 (Python Software Foundation 2020). The eye tracking and vehicle metrics used are each outlined in more detail below.

#### Eye tracking metrics

Time to first fixation towards the hazards was used to quantify how early drivers detected the hazards (Malone and Brünken 2020; Young et al. 2017). The shorter the time to first fixation towards the hazards, the earlier drivers detected both the materialised and non-materialised events. Consistent with previous HP studies with wearable eye trackers (Konstantopoulos et al. 2010; Urwyler et al. 2015), fixations were filtered with a minimum threshold of 100 ms. Fixations were calculated using the Velocity-Threshold Identification gaze filter (I-VT filter), with a threshold of 100 degrees/second (Salvucci and Goldberg 2000). Eye movements with a velocity below this threshold were classified as fixations, and those exceeding this threshold were classified as saccades.

To accurately quantify drivers’ fixation towards the hazards, dynamic Areas of Interests (AoI) were manually annotated using the Tobii Pro Lab (version 1.241). The AoIs represented the hazards, which were walking pedestrians and oncoming cars. Based on the visibility of the hazard in the simulation environment and previous research (e.g., Crundall et al. 2012), the event zone was operationalised as the section of the road spanning 100 m from the hazard, until the driver passed the hazard.

For events without any fixations, two reviewers were asked to conduct a blind review of the video recorded from the scene camera, to determine whether the drivers fixated on the hazardous elements (pedestrians/cars). This was done under the assumption that a potential error with the eye tracker calibration might have caused the gaze location to fall outside the defined bounding boxes for the hazard. A third reviewer was brought in to resolve the disagreements between the two reviewers, after which manual annotation was used for calculating the time to first fixation for these cases.

#### Vehicle metrics

The dependent variables derived from vehicular metrics are presented in Table 9 of the Appendix. Calculation of the dependent variables extracted from the vehicular metrics was made using the same event zones used for the eye tracking metrics (100 m prior to the event hazard). A data-driven approach, consistent with Louw et al. (2017), was used for action time (*t*_*action*_), with all driver reactions (braking and steering) plotted to set a threshold applicable for both manual driving and L2 automation. Drivers’ response to the events (*t*_*action*_) was operationalised as a brake pedal input higher than 5 Newton-metres (Nm) or a steering input of more than 2 degrees, in any direction.

### Data analysis

To answer the main research question, data was analysed using a generalised linear mixed model (GLMM) (Stroup 2012). All statistical models were conducted by having lighting of the external driving environment, level of driving automation, and the interaction between the two independent variables as fixed effects, and participant as the random effect, accounting for individual differences. All statistical analyses were conducted using R version 2024.04.2 (Rstudio Team 2024), and estimated marginal means (EMMs) were reported.

Separate models (for each event) were fitted as they were inherently differed in terms of their criticality, stimulus size and stimulus location (see Table 1), consistent with the approach used in previous studies (e.g., Borowsky et al. 2010). As the three metrics that were used to investigate drivers’ vehicular reaction towards the events (i.e., time to first relevant manoeuvres to materialised events, time to collision at reaction onset to materialised events and reaction to the non-materialised events) were on three isolated events (i.e., left pedestrian, right pedestrian, oncoming car), they were not directly comparable. Therefore, for these metrics, the alpha value of 0.05 was used as the criterion for indicating statistical significance and no alpha Bonferroni correction for multiple comparisons was needed.

The same approach was used for the time to first fixation metric, in which separate GLMMs were constructed for each of the six events. Although no direct comparisons were conducted between these models, three pairs of events (materialised and non-materialised left pedestrian, materialised and non-materialised right pedestrian; materialised and non-materialised oncoming car) could be reasonably considered to stem from the same conceptual “family” due to their similarity prior to materialisation of the event, respectively. As a precautionary measure to control for potential family-wise error within these pairs, an alpha Bonferroni correction for multiple testing was applied, adjusting the significance level (alpha-values) for this specific metric. For time to first fixation, the alpha value would be adjusted to *p* < 0.05/2, resulting *p* < 0.025 as the criterion for statistical significance.

As the interaction term is included in all the GLMM models, the model parameters generated from GLMM for the light condition and automation state reflect their effects within the reference category of the other variable (light condition reference: After Dark; automation state reference: L2 Automation). For this reason, post-hoc comparisons of EMMs were conducted to interpret the main effects of lighting of the external driving environment and level of driving automation. If any interaction effects are significant, post-hoc pairwise comparisons will be carried out with the Bonferroni correction.

In addition, due to the nature of the repeated-measures experimental design, participants were repeatedly exposed to the events. For this reason, separate statistical analyses, using GLMM, were conducted with event number as the fixed effect, and participant number as the random effect to examine any potential learning effects. This was used to examine whether repeatedly exposing drivers to events across experimental drives influenced their reaction. As preliminary analyses showed that event number yielded non-significant results for the majority of the dependent variables, and it was therefore excluded from the primary statistical models. This was done to maintain statistical parsimony.

From the 192 possible trials per event type (48 participants × 4 experimental drives), some were discarded due to technical issues with the driving simulator or eye tracker, or due to invalid trials (i.e., participants failed to follow the experimental protocol, or no reaction was captured). See Table 2 for the final number of trials included in the statistical models.

**Table 2:** Number of trials included in the statistical models

Materialisation status	Event type	Trials included in the statistical models	
		Eye tracking data	Vehicle data
Materialised	Left pedestrian	165	184
	Right pedestrian	164	185
	Oncoming car	165	189
Non-materialised	Left pedestrian	166	190
	Right pedestrian	166	190
	Oncoming car	166	190

The data and analysis codes are available on the repository: https://github.com/YeeThungLee/Hazard_Perception

## Results

Results of the eye tracking and vehicle data are reported in separate sections below.

### Time to first fixation to materialised events

To examine the influence of lighting of the external driving environment and level of driving automation on time to first fixation to materialised events, GLMMs were fitted to a Tweedie distribution (Brooks et al. 2017).

Results from the GLMM only showed a marginally significant main effect of lighting condition on time to first fixation for the materialised pedestrian from left event. Drivers fixated on the materialised pedestrian from left significantly earlier during daytime lighting (*EMMs* = 1.85, *SE* = 0.18) compared to after dark conditions (*EMMs* = 2.30, *SE* = 0.21; *z* = 2.234, *p* = 0.0255).

Time to first fixation to the materialised events was also found to be similar for the two levels of driving, suggesting no difference in hazard detection between manual driving and hands-off Level 2 automation (*p* > 0.025). There were also no significant interaction effects (*p* > 0.025) between the four experimental conditions for any of the materialised events (see Table 3).

**Table 3:** GLMM output for the time to first fixation to materialised events

Predictors	Coefficient (β)	SE	z	*p*	Exp (coefficient)	95% CI lower	95% CI higher
*1. Materialised left pedestrian event*							
Intercept	0.854	0.116	7.333	**< 0.001**	2.349	1.870	2.951
Light condition [day]	− 0.224	0.142	− 1.581	0.114	0.799	0.606	1.055
Automation state [manual driving]	− 0.045	0.134	− 0.335	0.738	0.956	0.734	1.244
Light condition [day] * automation state [manual driving]	0.012	0.195	0.063	0.950	1.012	0.690	1.485
*2. Materialised right pedestrian event*							
Intercept	0.531	0.153	3.466	**< 0.001**	1.701	1.260	2.297
Light condition [day]	0.084	0.184	0.456	0.648	1.088	0.758	1.561
Automation state [manual driving]	0.021	0.180	0.114	0.910	1.021	0.717	1.454
Light condition [day] * automation state [manual driving]	− 0.060	0.254	− 0.236	0.813	0.942	0.573	1.549
*3. Materialised oncoming car event*							
Intercept	0.239	0.195	1.224	0.221	1.270	0.866	1.861
Light condition [day]	− 0.420	0.239	− 1.756	0.079	0.657	0.411	1.050
Automation state [manual driving]	− 0.115	0.227	− 0.507	0.612	0.892	0.572	1.390
Light condition [day] * automation state [manual driving]	0.509	0.321	1.586	0.113	1.664	0.887	3.122

Probability distribution: Tweedie (Link function log). p-values in bold were statistically significant

### Time to first relevant manoeuvre to materialised events

To examine the effect of lighting and automation level on the time to first relevant manoeuvre to Materialised Events, a GLMM fitted to a gamma distribution was conducted for each event. Results from the GLMM showed that drivers’ time to first relevant manoeuvre to any of the materialised hazards was not different between the two lighting conditions (*p* > 0.05).

**Fig. 5:**
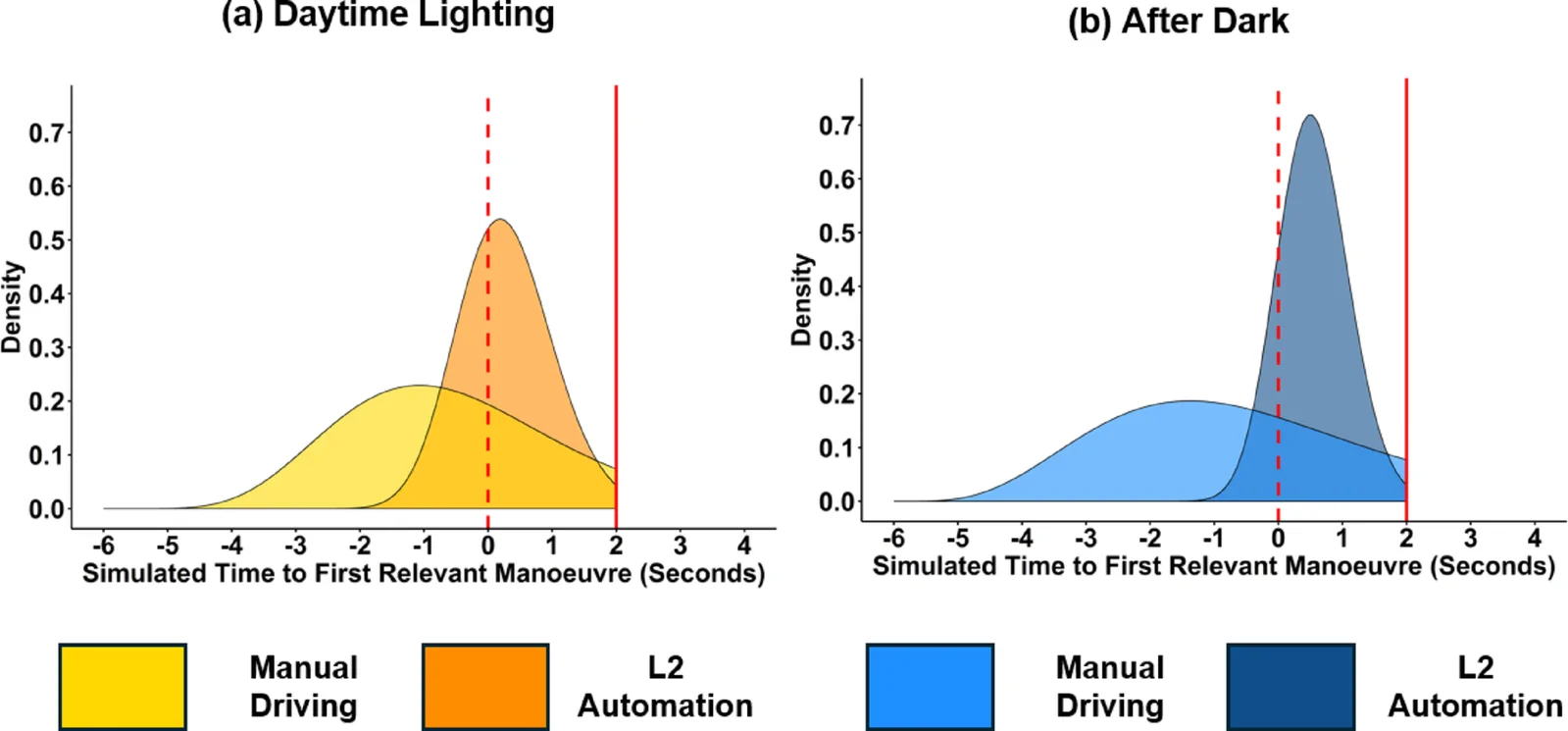
Simulated predicted distribution of time to first relevant manoeuvre to Materialised Left Pedestrian Event. Zero on the x-axis indicates onset of the materialised event, and 2 represents the maximum event TTC

Regarding the level of driving automation, the GLMM showed a significant main effect of level of driving automation on the time to first relevant manoeuvre to materialised events for the pedestrian from left (*z* = 4.472, *p* < 0.001), pedestrian from right (*z* = 3.301, *p* = 0.001), and oncoming car events (*z* = 3.446, *p* = 0.001). Participants reacted significantly earlier to the materialised events in the manual driving sections, compared to the L2 automation (see Table 4). The probability density function (PDF) plot illustrates the distribution of the simulated time to first relevant manoeuvres, estimated using the maximum likelihood estimation (MLE) method (Fig. 5). According to the distribution predictions from the model and the PDF plot, drivers were more proactive during the manual driving condition, responding earlier to the to the hazards. A similar pattern was seen for the pedestrian from right and oncoming car events. There were no significant interactions between the four experimental conditions for any of the materialised events (*p* > 0.05), (see Table 5).

**Table 4:** Descriptive data on time to first relevant manoeuvre to materialised events (seconds)

Event type	Level of driving automation	EMMs (SE)
Left pedestrian	Manual driving	− 0.58 (0.20)
	L2 automation	0.39 (0.23)
Right pedestrian	Manual driving	0.02 (0.15)
	L2 automation	0.66 (0.17)
Oncoming car	Manual driving	− 1.64 (0.24)
	L2 automation	− 0.59 (0.30)

The EMMs were transformed to their original scale for interpretability

**Table 5:** GLMM output for the time to first relevant manoeuvre to materialised events

Predictors	Coefficient (β)	SE	z	*p*	Exp (coefficient)	95% CI lower	95% CI higher
*1. Materialised left pedestrian event*							
Intercept	1.873	0.045	41.810	**< 0.001**	6.509	5.962	7.107
Light condition [day]	− 0.038	0.051	− 0.745	0.456	0.962	0.870	1.065
Automation state [manual driving]	− 0.182	0.051	− 3.543	**< 0.001**	0.834	0.754	0.922
Light condition [day] * automation state [manual driving]	0.037	0.073	0.511	0.610	1.038	0.900	1.197
*2. Materialised right pedestrian event*							
Intercept	1.522	0.049	31.130	**< 0.001**	4.581	4.162	5.041
Light condition [day]	0.033	0.062	0.532	0.595	1.034	0.915	1.168
Automation state [manual driving]	− 0.135	0.062	− 2.180	**0.029**	0.873	0.773	0.986
Light condition [day] * automation state [manual driving]	− 0.022	0.088	− 0.251	0.802	0.978	0.823	1.163
*3. Materialised oncoming car event*							
Intercept	1.655	0.071	23.297	**< 0.001**	5.233	4.553	6.014
Light condition [day]	0.067	0.089	0.753	0.452	1.069	0.898	1.272
Automation state [manual driving]	− 0.122	0.088	− 1.384	0.166	0.885	0.745	1.052
Light condition [day] * automation state [manual driving]	− 0.187	0.125	− 1.498	0.134	0.829	0.649	1.059

Probability distribution: Gamma (link function log). p-values in bold were statistically significant

### Time to collision at reaction onset to materialised events

To examine the effect of lighting and automation levels on the time to collision at reaction onset, a GLMM fitted to a gamma distribution was conducted for each event. The GLMM results showed that TTC for the three materialised events was not significantly affected by the lighting conditions (*p* > 0.05).

**Fig. 6:**
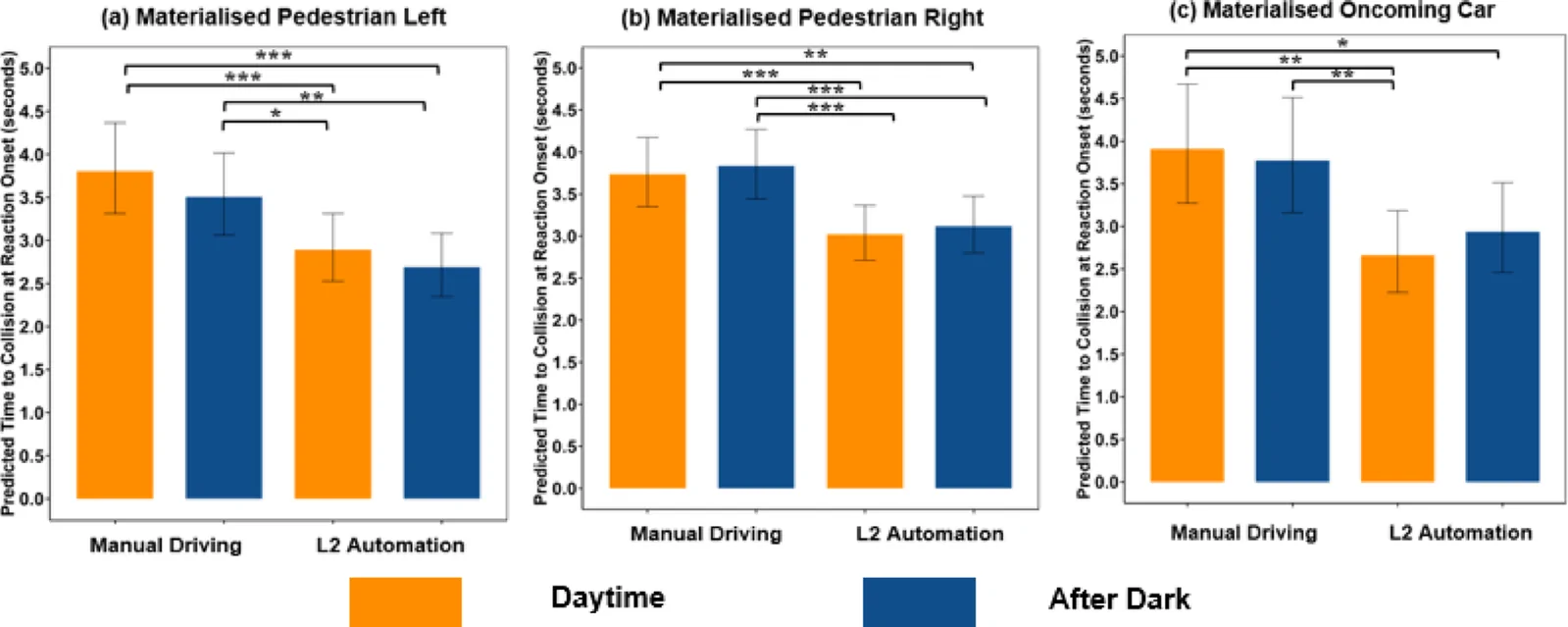
Bar plots showing the predicted means of time-to-collision for each experimental condition. Error bars showed 95% CI values from GLMM. *p_bonf_ < 0.05, **p_bonf_ < 0.01, ***p_bonf_ < 0.001

Regarding the level of driving automation, the GLMM showed a significant main effect of level of driving automation on the time to collision at reaction onset to the materialised pedestrian from left (*z* = -5.368, *p* < 0.001), materialised pedestrian from right (*z* = − 5.514, *p* < 0.001), and materialised oncoming car events (*z* = − 4.290, *p* < 0.001). Based on the distribution predictions from these three models (see Fig. 6), the time to collision at reaction onset was significantly higher in the manual driving condition, compared to that in L2 automation. There were no significant interactions between the four experimental conditions for any of the materialised events (*p* > 0.05), (see Table 6).

**Table 6:** GLMM output for the time to collision at reaction onset to Materialised Events

Predictors	Coefficient (β)	SE	z	*p*	Exp (Coefficient)	95% CI lower	95% CI higher
*1. Materialised left pedestrian event*							
Intercept	0.990	0.070	14.214	**< 0.001**	2.691	2.348	3.085
Light condition [day]	0.072	0.070	1.033	0.302	1.075	0.937	1.233
Automation state [manual driving]	0.264	0.070	3.768	**< 0.001**	1.303	1.135	1.495
Light condition [day] * automation state [manual driving]	0.009	0.099	0.094	0.925	1.009	0.831	1.226
*2. Materialised right pedestrian event*							
Intercept	1.137	0.055	20.534	**< 0.001**	3.118	2.797	3.475
Light condition [day]	− 0.032	0.053	− 0.593	0.553	0.969	0.873	1.076
Automation state [manual driving]	0.206	0.053	3.880	**< 0.001**	1.229	1.108	1.364
Light condition [day] * automation state [manual driving]	0.007	0.076	0.087	0.931	1.007	0.868	1.167
*3. Materialised oncoming car event*							
Intercept	1.078	0.091	11.843	**< 0.001**	2.938	2.458	3.512
Light condition [day]	− 0.099	0.106	− 0.941	0.347	0.906	0.736	1.114
Automation state [manual driving]	0.250	0.105	2.389	**0.017**	1.285	1.046	1.578
Light condition [day] * automation state [manual driving]	0.134	0.148	0.905	0.365	1.144	0.855	1.529

Probability distribution: Gamma (Link Function Log). p-values in bold were statistically significant

### Time to first fixation to non-materialised events

To examine the effect of lighting of the external driving environment and level of driving automation on time to first fixation towards the non-materialised hazards, GLMMs were fitted to a Tweedie distribution (Brooks et al. 2017).

The GLMM results only showed a significant main effect of lighting condition on the time to first fixation for the non-materialised pedestrian from left event, drivers fixated on the non-materialised pedestrian from left significantly earlier during daytime (*EMMs* = 1.52*, SE* = 0.14) compared to after dark conditions (*EMMs* = 2.16*, SE* = 0.19; *z* = 3.256*, p* = 0.001).

Time to first fixation was also found to be similar for the two levels of driving for all but the non-materialised pedestrian from left event. Here, the GLMM showed that drivers fixated on the non-materialised left pedestrian significantly earlier during the Level 2 conditions (*EMMs* = 1.58*, SE* = 0.15) compared to the manual driving conditions (*EMMs* = 2.07*, SE* = 0.18*; z* = − 2.468*, p* = 0.014). There were also no significant interaction effects (*p* > 0.025) between the four experimental conditions for any of the non-materialised events, (see Table 7).

**Table 7:** GLMM output for the time to first fixation to non-materialised events

Predictors	Coefficient (β)	SE	z	*p*	Exp (Coefficient)	95% CI lower	95% CI higher
*1. Non-materialised left pedestrian event*							
Intercept	0.586	0.120	4.897	**< .001**	1.797	1.421	2.273
Light condition [day]	− 0.254	0.162	− 1.570	0.116	0.775	0.565	1.065
Automation state [manual driving]	0.363	0.145	2.497	**0.013**	1.438	1.081	1.912
Light condition [day] * automation state [manual driving]	− 0.193	0.215	− 0.899	0.369	0.824	0.541	1.257
*2. Non-materialised right pedestrian event*							
Intercept	0.184	0.157	1.170	0.242	1.202	0.883	1.637
Light condition [day]	0.001	0.198	0.007	0.994	1.001	0.679	1.476
Automation state [manual driving]	− 0.050	0.194	− 0.258	0.797	0.951	0.650	1.392
Light condition [day] * automation state [manual driving]	− 0.061	0.275	− 0.222	0.824	0.941	0.549	1.612
*3. Non-materialised oncoming car event*							
Intercept	0.261	0.198	1.316	0.188	1.298	0.880	1.915
Light condition [day]	− 0.452	0.251	− 1.798	0.072	0.637	0.389	1.042
Automation state [manual driving]	− 0.172	0.239	− 0.720	0.472	0.842	0.527	1.346
Light condition [day] * automation state [manual driving]	0.279	0.343	0.813	0.416	1.321	0.675	2.587

Probability distribution: Tweedie (Link Function Log). p-values in bold were statistically significant

### Reaction to the non-materialised events

To examine whether the lighting of the external driving environment and level of driving automation influenced reaction to the non-materialised events, a GLMM fitted with a binomial distribution—logit link function (0 = no reaction, 1 = reacted) was conducted for all three non-materialised events.

Results showed that lighting of the driving environment only influenced response to the non-materialised pedestrian from left event, with a higher likelihood of reacting to event during daytime conditions (*z* = − 2.371, *p* = 0.018).

**Fig. 7:**
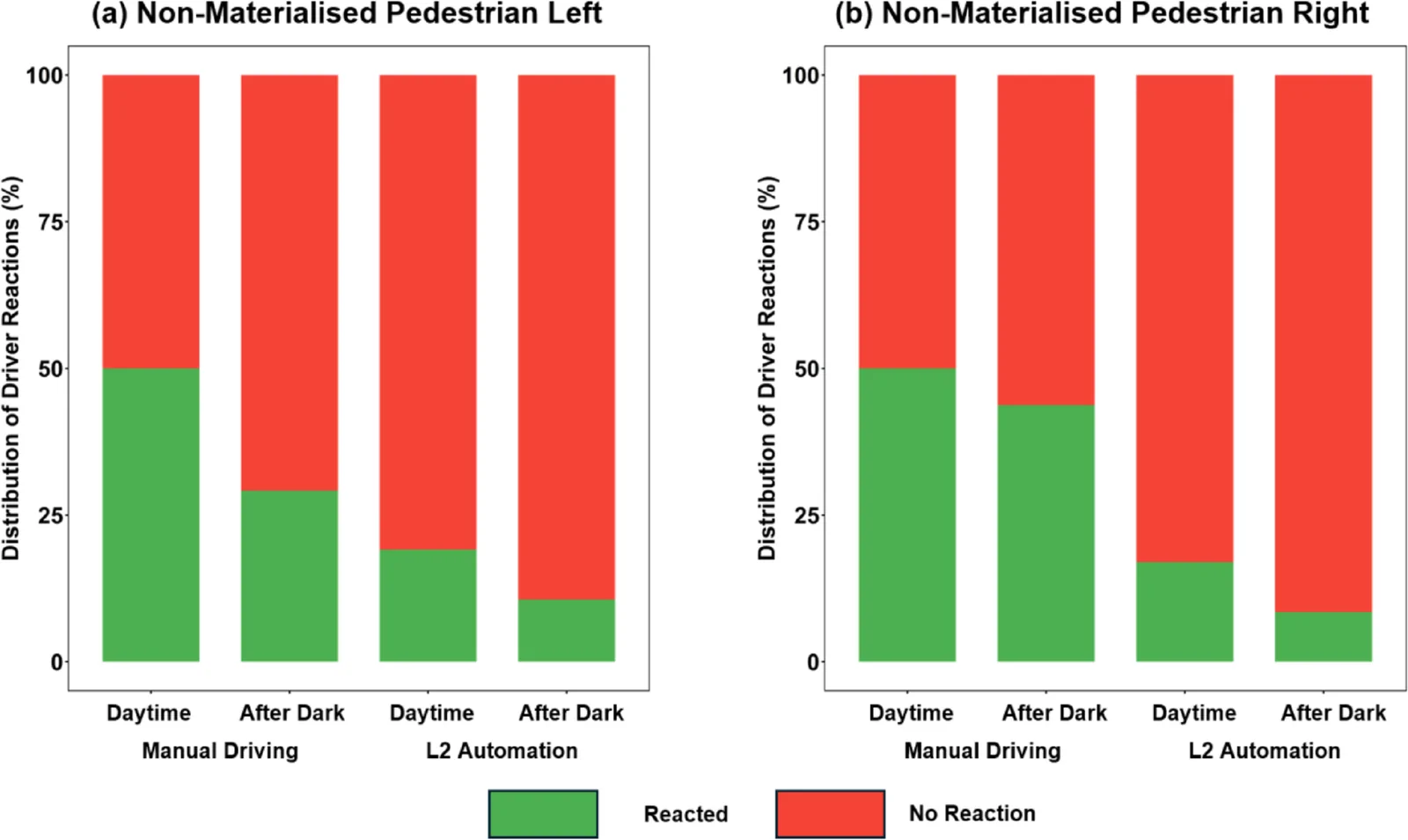
Stacked bar plots showing the distribution of driver reactions for each experimental condition

Regarding the level of driving automation, the GLMM showed a significant main effect of level of driving automation on response to the non-materialised pedestrian from left (*z* = − 3.817*, p* < 0.001) and non-materialised pedestrian from right events (*z* = − 4.772*, p* < 0.001). As shown in Fig. 7, participants had significantly higher likelihood of reacting to the events during manual driving, compared to the L2 automation conditions. However, this difference was not seen for the oncoming car event (*z* = − 1.805*, p* = 0.071). None of the interaction effects were found to be significant (*p* > 0.05), (see Table 8).

**Table 8:** GLMM output for the reaction to Non-Materialised Events

Predictors	Coefficient (β)	SE	z	*p*	Odds ratio	95% CI lower	95% CI higher
*1. Non-materialised left pedestrian event*							
Intercept	− 2.743	0.624	− 4.398	**< 0.001**	0.064	0.019	0.219
Light condition [day]	0.822	0.670	1.227	0.220	2.274	0.612	8.449
Automation state [manual driving]	1.539	0.652	2.362	**0.018**	4.661	1.299	16.721
Light condition [day] * automation state [manual driving]	0.395	0.836	0.472	0.637	1.484	0.288	7.635
*2. Non-materialised right pedestrian event*							
Intercept	− 2.968	0.658	− 4.512	**< 0.001**	0.051	0.014	0.187
Light condition [day]	0.931	0.703	1.324	0.186	2.536	0.639	10.059
Automation state [manual driving]	2.637	0.701	3.761	**< 0.001**	13.966	3.534	55.189
Light condition [day] * automation state [manual driving]	− 0.593	0.846	− 0.701	0.0483	0.553	0.105	2.899
*3. Non-materialised oncoming car event*							
Intercept	0.721	0.336	2.150	**0.032**	2.057	1.066	3.971
Light condition [day]	− 0.590	0.445	− 1.326	0.185	0.555	0.232	1.326
Automation state [manual driving]	0.346	0.462	0.748	0.454	1.413	0.571	3.499
Light condition [day] * automation state [manual driving]	0.480	0.645	0.744	0.457	1.615	0.456	5.720

Probability distribution: Binomial (Link Function Logit). No reaction was coded as 0; Reaction was coded as 1. p-values in bold were statistically significant

### Learning effects

The GLMM results only showed a significant main effect of event number on the dependent variables time to first fixation to the materialised oncoming car (*p* = 0.017), and the likelihood of reacting to the non-materialised left pedestrian events (*p* = 0.021). No other main effects were found. Overall, as event number increased, participants showed a significantly faster time to first fixation to the materialised oncoming car event, and were significantly less likely to react to the non-materialised left pedestrian (see Tables 10, 11, 12, 13, 14in the Appendix).

## Discussion

The aim of this driving simulator study was to compare drivers’ HP in two different lighting conditions (daytime and after dark), during manual and hands-off Level 2 automated driving. Performance was compared in terms of time to first fixation to the hazard and driver response to six of the events.

After applying the Bonferroni-adjusted significance threshold, results showed a significant effect of lighting on the time to first fixation to non-materialised pedestrians approaching from the left (*p* = 0.001), and marginally significant for the materialised pedestrian approaching from the left *(p* = 0.0255)*.* In line with previous studies (Evans et al. 2022; Garay et al. 2004; Garay-Vega et al. 2007), drivers detected these hazards earlier during daytime, than after dark. This may be due to the diminished visibility in the after dark conditions, which reduces drivers’ gaze dispersion and sight distance for peripheral hazards (Grüner and Ansorge 2017). It has previously been argued that humans’ visual performance deteriorates, especially in contrast sensitivity and visual acuity, in reduced illumination conditions such as driving in after dark environment (Wood 2020), which further supports our findings.

Drivers’ vehicular reaction towards the non-materialised left pedestrian continued to be better in daytime, compared to after dark conditions, as demonstrated by a significantly higher probability of initiating a vehicular manoeuvre, even when that pedestrian did not ultimately cross the road. This is likely because hazards were detected earlier in the daytime, compared to after dark conditions.

There was no effect of lighting condition for the pedestrian from right and oncoming car events, which may be due to the differences in the choreography of the scenarios. Specifically, the faster walking speed of the right pedestrian and the saliency of the turn indicator on the oncoming car (see Table 1) may have captured drivers’ attention, regardless of the lighting condition of the driving environment. Based on the Signal Detection Theory (SDT; Green and Swets 1966; Swets 1998; Macmillan and Creelman 2005; Makowski 2018), more salient stimuli, such as those that stand out due to brightness or motion, are more likely to be detected in environments with reduced visibility.

In terms of time to first relevant manoeuvres and TTC for the three materialised hazards, no significant differences were found between daytime and after dark conditions. To the best of our knowledge, this study is one of the first to measure drivers’ actual response to hazards with real driving-based actions. This approach provides a more ecologically valid and direct measure of the complex decision-making processes involved in braking or steering for hazard avoidance, rather than relying on the commonly used button-pressing responses used in prior studies. Previous studies report earlier detection of hazards in daytime driving environments (Evans et al. 2022; Garay et al. 2004; Garay-Vega et al. 2007) as also shown for the detection of our pedestrian approaching from left events. However, it seems that this earlier detection does not necessarily translate to a quicker vehicular reaction to the hazards. These findings contribute to the current state of the art, by highlighting a disconnection between hazard detection and subsequent vehicular reaction towards hazards, suggesting that while visibility improves detection, lighting of the environment does not affect response time.

In terms of the effect of level of driving automation on performance, results showed that (except for the non-materialised pedestrian from left event) the time to first fixation to the hazards was the same for manual driving and L2 automation, which is in line with results of previous studies in this context (Greenlee et al. 2024; Samuel et al. 2020; Zangi et al. 2022). The results also showed that no significant interaction effect was found on any of the metrics, suggesting that the influence of automation was not modulated by the lighting conditions, which rejected our hypothesis. It is possible that the reduced visibility in after dark driving environments in the present study was not detrimental enough to induce any observed effect of compensatory behaviours as we had hypothesised. Authors from previous studies have argued that removing the effort required in the motor-control-loop of manual driving provides more attentional resources for HP in L2 automation (Mackenzie and Harris 2015; Mars and Navarro 2012; Navarro et al. 2016). However, this prediction was not supported by the results of our study. This may be because the attentional demand associated with the detection of only one hazardous event at a time may have been relatively straight forward during a well-rehearsed and automatised manual driving task (Engström et al. 2017; McKnight and Adams 1970), still providing adequate resources for hazard detection. Further work involving detection of hazards in a more complex driving environment is, therefore, required to investigate this discrepancy between our study and previous work.

The significant effect of level of driving automation on the time to first fixation for the non-materialised left pedestrian event should be interpreted with caution. Further inspection of the data showed that drivers’ first fixation typically occurred before the pedestrian arrived at the edge of the road. At this point, the visual cues provided by the materialised and non-materialised version of the left pedestrian events were identical, including the pedestrian’s walking speed. Therefore, we would expect consistent results for the effect of level of driving automation on their time to first fixation, regardless of whether the event ultimately materialised. The isolated significant result observed for non-materialised left pedestrian events could be a potential Type 1 error, rather than the effect of the experimental manipulation (Field 2017), despite our effort in adopting a stricter alpha value (0.025) after Bonferroni correction for this metric.

The effect of the level of driving automation on drivers’ time to first relevant manoeuvres and TTC in response to the three materialised events is consistent with previous studies (Blommer et al. 2017; Eriksson and Stanton 2017; Gold et al. 2013), with their first relevant manoeuvre being significantly later and their TTC being significantly lower in L2 automation, compared to manual driving. The majority of drivers were found to be proactive in their response to hazards during manual driving, as opposed to providing more reactive responses during L2 automation. Results obtained from the drivers’ reaction to non-materialised events also confirmed these findings. As highlighted by Louw et al. (2017), drivers in automated driving tend to initiate collision avoidance manoeuvres based on the perceived criticality of the hazardous events. As shown in Fig. 5, the majority of drivers in L2 automation only reacted after the events escalated into a more critical and imminent stage, such as when the pedestrian started to step onto the road. Given that the level of driving automation significantly influences response to the hazards but not the time to first fixation, these findings suggest that drivers in L2 automation reacted to the developing criticality of the hazardous events, rather than after initial detection of the latent hazard. One plausible explanation could be that drivers had mismatched expectations about the capabilities of the automated system, expecting it to respond, or waiting until the very last second before responding. This is similar to results found in previous studies (Victor et al. 2018), with explicit explanation that the automated system in the study was not capable of detecting or responding to any on-road hazards. Another plausible explanation is that the L2 automation used in the present study was hands-off, requiring additional time to take over manual control. A previous study suggested that simply having hands-off L2 could further prolonged the time needed to react to a silent automation failure (Hussain et al. 2025). While the study’s results might not be directly transferable to hands-on L2 currently on the market, it still provides valuable insights into the effect of L2 automation (i.e., supervisor role) on their subsequent ability to react to hazards when not prompted.

However, at least for two of the events, participants’ response was found to improve over time. GLMM with event number as the fixed effect variable showed a faster time to fixation to the materialised oncoming car and a lower probability of manoeuvre initiation to the non-materialised pedestrian from left events. This suggests that both events were easier to recognise after first exposure, perhaps due to the saliency of the turn indicator and the conduct of the non-materialised pedestrian.

### Limitations and future work

One main limitation of this study was the use of a driving simulator for understanding HP at night. While driving simulators provide a controlled and safe environment for such HP studies, they may not reflect drivers’ actual driving behaviour in real-life settings, particularly for studying response in night time conditions. Future work may benefit from conducting this study during the night time hours, to assess the effect of circadian rhythms on performance, as well as investigating HP during L2 automation on the road, to boost ecological validity.

## Conclusion

To conclude, the present study provides new insights on how drivers’ hazard perception might be affected by the lighting of the driving environment and the presence of automation. The lighting of the driving environment significantly impacted drivers’ initial detection of the hazards, particularly for the pedestrians approaching from the left. In contrast, lighting of the driving environment did not influence drivers’ response execution towards the hazards. Regarding the level of driving automation, changes in drivers’ role from an operator to a supervisor of the vehicle in the hands-off L2 automated driving conditions influenced their reaction towards hazards. The majority of drivers were proactive and responded early to hazards during manual driving, as opposed to being more reactive during L2 automated driving. These results highlight the need of considering the context of the driving environment, such as lighting conditions and level of driving automation when designing systems or protocols that aim to support hazard perception and timely driver response.

## Data Availability

The data and analysis codes are available on the repository: https://github.com/YeeThungLee/Hazard_Perception.

## Appendix

See Tables

**Table 9:** Metrics used to evaluate hazard avoidance

Metrics used	Events	Rationale
1. Time to first relevant manoeuvre (s)	Materialised hazards only	To quantify how early the driver reacted to avoid the hazard relative to the hazard materialisation onset Hazard materialisation onset was operationalised as the timestamp where the materialised event started to develop into an actual hazardous event (materialises)—i.e. pedestrian starts stepping out of the road/car starts to turn Time to first relevant manoeuvre was calculated by subtracting the timestamp of hazard materialisation onset from *t*_*action*_ A negative value indicated that the driver reacted before the hazard materialised, whereas a positive value indicated that the driver reacted after the hazard materialised
2. Time to collision at reaction onset (TTC) (s)	Materialised hazards only	TTC was calculated as the longitudinal distance (m) between the ego-vehicle and the event hazard divided by vehicle’s speed during the reaction onset To examine the criticality of the unfolding hazard events when drivers first reacted The lower the TTC at reaction onset, the more critical the unfolding hazard events
3. Reaction to the non-materialised hazards	Non-materialised hazards only	Calculated as a categorical variable, indicating the presence of a reaction input (as defined above) within the event zone To examine whether drivers opted to react to potential hazard (non-materialised hazard)

9,

**Table 10:** GLMM output for the effect of event number on time to first fixation to Materialised Events

Predictors	Coefficient (β)	SE	z	*p*	Exp (coefficient)	95% CI lower	95% CI higher
*1. Materialised left pedestrian event*							
Intercept	0.832	0.118	7.047	**< 0.001**	2.229	1.824	2.897
Event number	− 0.009	0.007	− 1.186	0.236	0.992	0.978	1.006
*2. Materialised right pedestrian event*							
Intercept	0.621	0.149	4.169	**< 0.001**	1.861	1.390	2.492
Event number	− 0.004	0.009	− 0.478	0.633	0.996	0.978	1.014
*3. Materialised oncoming car event*							
Intercept	0.456	0.198	2.300	**0.021**	1.579	1.070	2.329
Event number	− 0.029	0.012	− 2.378	**0.017**	0.971	0.948	0.995

Probability distribution: Tweedie (Link Function Log)

10,

**Table 11:** LMM output for the effect of event number on time to first relevant manoeuvre to materialised events

Predictors	Coefficient (β)	SE	Z	*p*	Exp (coefficient)	95% CI lower	95% CI higher
*1. Materialised left pedestrian event*							
Intercept	1.798	0.046	39.353	**< 0.001**	6.035	5.518	6.600
Event number	− 0.002	0.003	− 0.594	0.552	0.998	0.993	1.004
*2. Materialised right pedestrian event*							
Intercept	1.543	0.049	31.387	**< 0.001**	4.678	4.248	5.151
Event number	− 0.006	0.003	− 1.856	0.064	0.994	0.988	1.000
*3. Materialised oncoming car event*							
Intercept	1.668	0.148	11.281	**< 0.001**	5.301	3.967	7.082
Event number	− 0.005	0.010	− 0.453	0.651	0.995	0.975	1.016

Probability distribution: Gamma (Link Function Log)

11,

**Table 12:** GLMM output for the effect of event number on time to collision at reaction onset to Materialised Events

Predictors	Coefficient (β)	SE	Z	*p*	Exp (coefficient)	95% CI lower	95% CI higher
*1. Materialised left pedestrian event*							
Intercept	1.175	0.076	15.454	**< 0.001**	3.237	2.789	3.758
Event number	− 0.001	0.004	− 0.119	0.905	1.000	0.992	1.007
*2. Materialised right pedestrian event*							
Intercept	1.159	0.058	19.858	**< 0.001**	3.187	2.842	3.573
Event number	0.006	0.003	1.919	0.055	1.006	1.000	1.012
*3. Materialised oncoming car event*							
Intercept	1.082	0.155	6.975	**< 0.001**	2.951	2.177	4.000
Event number	0.014	0.011	1.305	0.192	1.015	0.993	1.037

Probability distribution: Gamma (Link Function Log)

12,

**Table 13:** GLMM output for the effect of event number on time to first fixation to non-materialised events

Predictors	Coefficient (β)	SE	z	*p*	Exp (coefficient)	95% CI lower	95% CI higher
*1. Non-materialised left pedestrian event*							
Intercept	0.640	0.125	5.118	**< 0.001**	1.896	1.484	2.422
Event number	− 0.001	0.008	− 0.083	0.934	0.999	0.983	1.016
*2. Non-materialised right pedestrian event*							
Intercept	0.097	0.161	0.603	0.546	1.102	0.804	1.509
Event number	0.004	0.010	0.371	0.711	1.004	0.984	1.024
*3. Non-materialised oncoming car event*							
Intercept	0.086	0.217	0.395	0.693	1.090	0.712	1.667
Event number	− 0.004	0.013	− 0.306	0.760	0.996	0.971	1.022

Probability distribution: Tweedie (Link Function Log)

13,

**Table 14:** GLMM output for the effect of event number on reaction to the non-materialised events

Predictors	Coefficient (β)	SE	z	*p*	Odds ratio	95% CI lower	95% CI higher
*1. Non-materialised left pedestrian event*							
Intercept	− 0.442	0.386	− 1.147	0.252	0.643	0.302	1.369
Event number	− 0.063	0.027	− 2.317	**0.021**	0.939	0.891	0.990
*2. Non-materialised right pedestrian event*							
Intercept	− 1.183	0.374	− 3.160	**0.002**	0.306	0.147	0.638
Event number	0.018	0.024	0.770	0.441	1.019	0.972	1.067
*3. Non-materialised oncoming car event*							
Intercept	0.894	0.359	2.489	**0.013**	2.445	1.209	4.943
Event number	− 0.015	0.023	− 0.652	0.515	0.958	0.941	1.031

Probability distribution: Binomial (logit link function). No reaction was coded as 0; Reaction was coded as 1

^14^.
